# Combined Taurine, Epigallocatechin Gallate and Genistein Therapy Reduces HSC-T6 Cell Proliferation and Modulates the Expression of Fibrogenic Factors

**DOI:** 10.3390/ijms141020543

**Published:** 2013-10-14

**Authors:** Yan Li, Ying Luo, Xuerong Zhang, Xing Lin, Min He, Ming Liao

**Affiliations:** 1 Guangxi University Library, Guangxi University, Nanning 530004, Guangxi, China; E-Mail: yan_01091@sina.com; 2 Key Laboratory Joint Established by Ministry of Education and Guangxi, Medical Scientific Research Center of Guangxi Medical University, Nanning 530021, Guangxi, China; E-Mails: luoyinggx@163.com (Y.L.); xrongzhang@163.com (X.Z.); linxinggx@163.com (X.L.); hminmu@163.com (M.H.)

**Keywords:** taurine, epigallocatechin gallate, fibrosis-related genes, hepatic stellate cells, liver fibrosis, proteomics

## Abstract

Hepatic fibrogenesis involves the activation of hepatic stellate cells (HSCs), which synthesize excess extracellular matrix and contribute to the development of liver fibrosis. In a prior study we tested the effect of combined treatment with taurine, epigallocatechin gallate and genistein on the development of alcohol-induced liver fibrosis *in vitro*. In this study, the biological activity of the combination of these molecules was assessed by measuring its effect on cell proliferation, fibrosis-related gene expression, and proteomic expression profiling in the activated HSC cell line, HSC-T6. HSC-T6 cells were incubated with different concentrations of the drug combination taurine, epigallocatechin gallate and genistein. Cell proliferation was evaluated by MTT assay. Transforming growth factor β1 (*TGF-β1*), collagen type I (*Col-I*), matrix metalloproteinase-2 (*MMP-2*), and tissue inhibitor of metalloproteinases 1 and 2 (*TIMP-1* and *TIMP-2*) mRNA were analyzed by semi-quantitative reverse-transcription PCR. Proteomic profiling of HSC-T6 cells was also performed by SELDI-TOF-MS. Combined drug treatment significantly inhibited cell proliferation and *TGF-β1*, *Col-I*, *TIMP-1* and *TIMP-2* mRNA expression in activated HSC-T6 cells, while the expression of *MMP-2* mRNA increased. A total of 176 protein *m/z* peaks were identified. The intensities of 10 protein peaks were downregulated and two protein peaks were upregulated in HSC-T6 cells after combined drug treatment. In conclusion, combined drug treatment with taurine, epigallocatechin gallate and genistein can inhibit HSC proliferation, and impact fibrosis-related gene and protein expression. The antifibrotic effects of this drug combination may be due to its effects on the expression of fibrogenic genes.

## 1. Introduction

Liver fibrosis is a reversible wound-healing response to either acute or chronic cellular injury that reflects a balance between liver repair and scar formation [1]. In the course of fibrogenesis, hepatic stellate cells (HSCs) undergo activation, transdifferentiate into a myofibroblast-like phenotype, and proliferate and synthesize excess extra-cellular matrix (ECM) components, particularly collagen [2,3]. Therefore, it is important to identify agents that might limit the proliferation of HSCs or their ability to synthesize excess ECM.

It is known that liver fibrosis can be triggered by a series of insults, involving a complex disease process that requires multiple therapies [4]. It is obvious that monotherapy with either tyrosine kinase inhibitors or antioxidants has limited effects [5–7]. Furthermore, the use of a single drug at a high dose can cause overwhelming side effects [8]. As such, research into the identification of more effective therapies to treat hepatic fibrosis with fewer side effects is warranted. In this regard, combination therapies with multiple drugs are thought to be superior to monotherapy because of their additive or synergistic effects and relief from side effects [9,10]. Combination therapies employ multiple drugs that can target different sites of action and molecules, as well as intervening at different phases of fibrogenesis, potentially providing a powerful therapy to treat liver fibrosis. It has been reported that taurine, epigallocatechin gallate (EGCG) and genistein, which have different therapeutic targets, are effective against liver fibrosis. Taurine can promote apoptosis in HSCs by inhibiting the expression of TGF-β1 and thereby blocking the TGF-β1/Smad pathway [11–13]. It has also been reported that EGCG possesses remarkable antioxidant properties that can suppress the secretion of collagen [14]. Lastly, genistein, a tyrosine kinase inhibitor, can inhibit the proliferation of liver sinusoidal endothelial cells [15]. We hypothesized that combined treatment with taurine, EGCG and genistein may be an effective therapy for the treatment of liver fibrosis. Previously, we reported that combined therapy with taurine, EGCG and genistein had an obvious protective effect against alcohol-induced liver fibrosis in rats by suppressing the serum levels of fibrosis markers and hepatic hydroxyproline content, as well as inhibiting hepatic collagen deposition and protein expression of B-cell lymphoma 2, α-smooth muscle actin, TGF-β1, and others against decapentaplegic homolog 3 (SMAD-3) [16]. In the current study, we evaluated the mechanistic effect of combined taurine, EGCG and genistein therapy on hepatic fibrosis using an *in vitro* HSC model. We believe this research will provide new insights into the use of combined therapies to treat hepatic fibrosis.

## 2. Results

### 2.1. Effect of Combined Drug Treatment on Cell Proliferation

**Figure 1:**
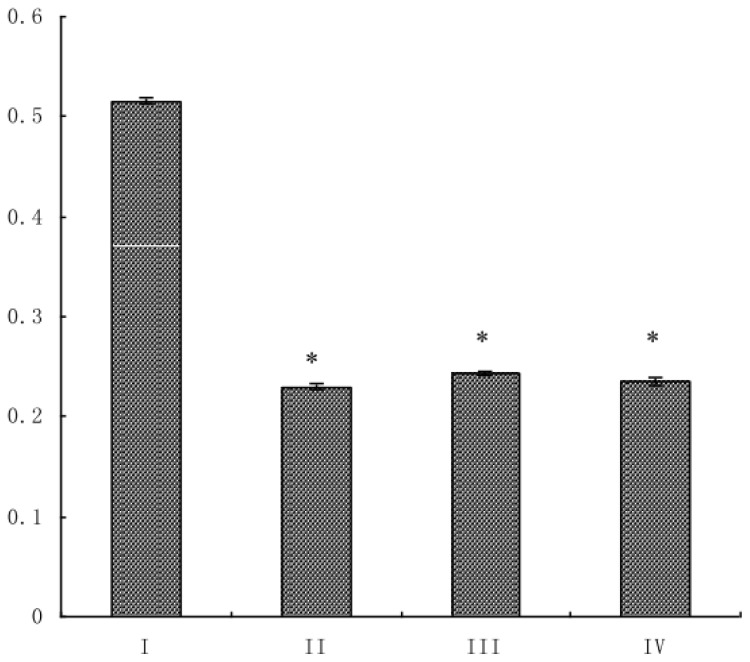
Proliferation of HSC-T6 cells (hepatic stellate cells) treated with a combination of taurine, epigallocatechin gallate (EGCG) and genistein. HSC-T6 cell proliferation was measured by MTT assay. The effect of varying doses of taurine, EGCG and genistein were compared to (I) untreated control cells; or cells treated with (II) 0.015 mg/mL taurine, 0.0175 mg/mL EGCG and 0.0035 mg/mL genistein; (III) 0.03 mg/mL taurine, 0.035 mg/mL EGCG and 0.007 mg/mL genistein; or (IV) 0.06 mg/mL taurine, 0.07 mg/mL EGCG and 0.014 mg/mL genistein. Data are presented as the mean ± S.E.; * *p* <0.05 *vs.* group I (untreated control).

The anti-proliferative effect of combined taurine, EGCG and genistein treatment in HSC-T6 cells was determined by measuring cell viability using MTT assay. After treatment with different doses of the drug combination, the proliferation of all treated groups was significantly reduced compared to control HSCs. As shown in Figure 1, HSC-T6 cell proliferation was remarkably inhibited by the drug combinations.

### 2.2. Effects of Combined Drug Rreatment on TGF-β1, Col-I, TIMP-1, TIMP-2 and MMP-2 mRNA Expression in HSC-T6 Cells

**Figure 2:**
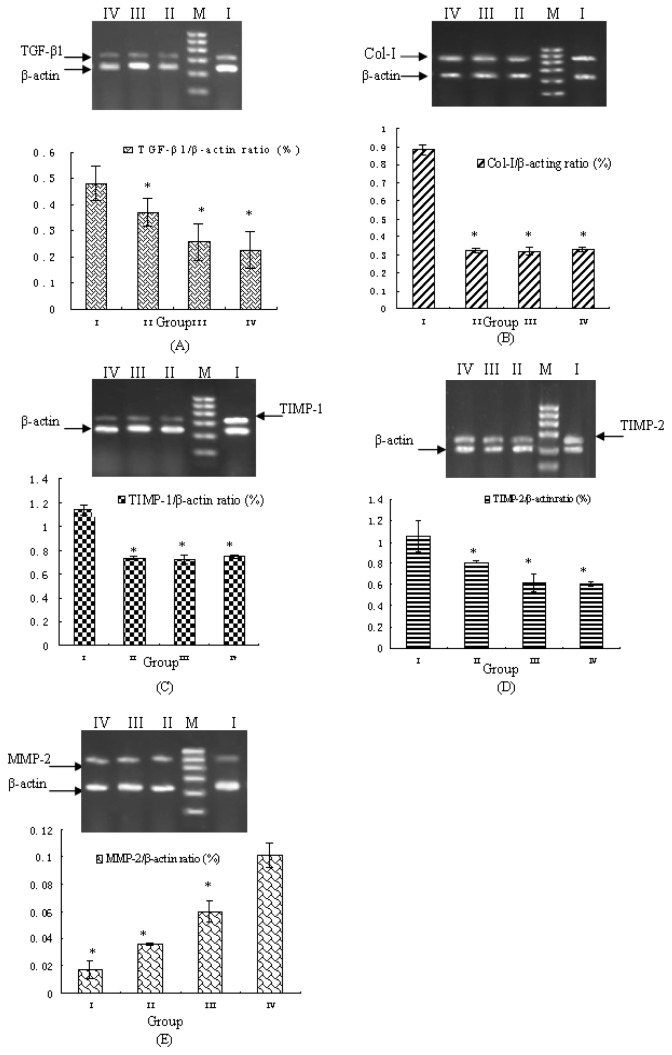
The mRNA expression levels of genes associated with hepatic fibrogenesis in HSC-T6 cells treated with a combination of taurine, EGCG and genistein. Semi-quantitative PCR data are shown for cells treated with a combination of taurine, EGCG and genistein in varying doses. The relative mRNA expression levels of *TGF-β1* (**A**), *Col-I* (**B**), *TIMP-1* (**C**), *TIMP-2* (**D**), and *MMP-2* (**E**) are presented; upper panels shown representative pictures of agarose gels and the lower panels show relative quantification of mRNA expression levels in (I) untreated control cells; or cells treated with (II) 0.015 mg/mL taurine, 0.0175 mg/mL EGCG and 0.0035 mg/mL genistein; (III) 0.03 mg/mL taurine, 0.035 mg/mL EGCG and 0.007 mg/mL genistein; or (IV) 0.06 mg/mL taurine, 0.07 mg/mL EGCG and 0.014 mg/mL genistein; (M) marker. Data are presented as the mean ± S.E.; * *p* <0.05 *vs.* group I (untreated control).

The mRNA expression levels of *TGF-β1*, *Col-I*, *TIMP-1*, *TIMP-2* and *MMP-2* were measured by PCR in HSC-T6 cells treated in combination with taurine, EGCG and genistein. As shown in Figure 2, compared with the control group, the medium (group II) and high dosage (group III) combinations significantly downregulated the mRNA expression levels of *TGF-β1*, *Col-I*, *TIMP-1* and *TIMP-2*. On the other hand, the expression level of *MMP-2* significantly increased in the treated cells (Figure 2E).

### 2.3. Identification of Fibrosis-Associated Proteins in the HSC-T6 Proteome

**Figure 3:**
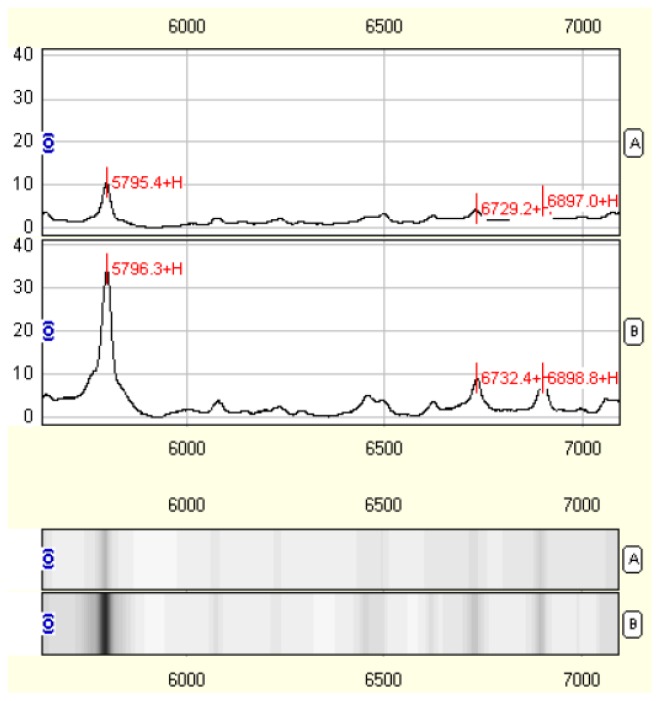
Proteomic analysis of HSC-T6 cells treated with a combination of taurine, EGCG and genistein. We performed a proteomic analysis of taurine, EGCG and genistein-treated HSC-T6 cells (**A**) and control HSC-T6 cells (**B**). Representative protein peaks and gel views of the peaks which positively correlated with fibrosis with a *m*/*z* 5798.33, 6735.19 and 6896.73 are shown.

In order to detect fibrosis-associated proteins in treated HSC-T6 cells, their proteome was analyzed by SELDI-TOF-MS. Note, for this analysis we only compared the cells in group I and group III. A total of 176 proteomic peaks were generated, 12 of these peaks had mean intensity values that differed significantly between control and treated cells. Ten protein peaks were lower in the treated cells and had mass-to-charge (*m*/*z*) ratios of 2897.76, 5361.62, 5562.06, 5645.97, 5798.33, 6141.22, 6499.74, 6623.38, 6735.19 and 6896.73. Two protein peaks, with a *m*/*z* of 4656.91 and 5078.35, were higher in treated cells (Table 1 and Figure 3).

The identity of these 12 proteomic peaks was determined using Tagldent software and the Swiss-Prot proteome database (http://www.expasy.org/proteomics/). Four of the peaks (*m*/*z* 6896.73, 6623.38, 5798.33 and 4656.91) are thought to be potentially related to the whey acidic protein four disulfide core domain 15A (WFDC 15A), luteinizing hormone release inhibiting factor (LHRIF), natriuretic peptide-53, and cytochrome c oxidase subunit 8B, respectively (Table 2). The other eight peaks could not be identified.

## 3. Discussion

A key discovery in understanding the mechanisms underlying hepatic fibrosis has been the identification of HSCs as the primary effector cell in the pathogenic disease process, as they orchestrate the deposition of ECM in the normal and fibrotic liver [17]. Activated HSCs are known to be the main source of ECM when liver fibrosis occurs, and an effective way to reverse liver fibrosis is to inhibit the proliferation of HSCs [18,19]. In our study, combined treatment with taurine, EGCG and genistein remarkably suppressed HSC-T6 cell proliferation, leading us to conclude that this combined treatment regime could inhibit hepatic fibrosis by inhibiting the proliferation of activated HSCs.

Fibrogenesis, which results from an imbalance between the synthesis and degradation of ECM, contributes to the development of liver fibrosis [20]. TGFβ1 is the most potent fibrogenic cytokine in the liver; its expression increases during fibrogenesis and it is the dominant stimulus which induces HSCs to increase ECM synthesis [21]. Stimulation of activated HSCs by TGF-β1 is believed to be the key fibrogenic response in liver fibrosis [22]; strategies aimed at disrupting TGF-β1 expression are a key approach in the prevention and treatment of liver fibrosis [23]. The degradation of ECM is primarily regulated by matrix metalloproteinases (MMPs), the activity of which can be suppressed by TIMPs [24]. TIMP-1 has been demonstrated to suppress apoptosis in HSCs and reduce MMP activity [25], which strongly promoted the development of liver fibrosis in a transgenic mouse model [26]. MMP-2 is produced by HSCs and plays an important role in liver fibrogenesis, and MMP-2 expression is elevated in the anti-fibrotic liver [27,28]. Additionally, during hepatic fibrogenesis, collagen proliferation (primarily collagen types 1 and 3), accounts for 50% of the total proteins in the fibrotic liver [29], and collagens are the main components of the ECM. Therefore, collagen type 1 is an important parameter which reflects collagen metabolism in the liver. The main collagen-producing cells in the liver are HSCs, which have an increased capacity for collagen synthesis when activated [30].

In this study, we found that treatment of HSC-T6 cells with a combination of taurine, EGCG and genistein resulted in a marked downregulation in the mRNA levels of profibrogenic factors, including *TGF-β1*, *TIMP-1*, *TIMP-2* and *Col-I*. On the other hand, expression of the antifibrogenic gene *MMP2* significantly increased in treated cells. These results indicate that combined taurine, EGCG and genistein treatment may induce ECM degradation in the liver by decreasing the expression of TGF-β1, TIMPs and collagen I, and elevating MMP-2 expression, which combine to produce an anti-fibrogenic effect.

In our analysis of the proteome of HSC-T6 cells treated with taurine, EGCG and genistein, we identified 12 differentially expressed proteomic peaks. Four of these peaks are thought to be potentially related to WFDC 15A, LHRIF, cytochrome c oxidase subunit 8B, and natriuretic peptide-53, respectively. The WFDC domain is present in some small serine proteinase inhibitors, and proteins in this family have a biological role that is closely related to the innate immune system, inflammation and the inhibition of cell growth [31]. LHRIF is involved in cellular signal transduction and is a regulator of cell proliferation [32,33]. The cytochrome c oxidase subunit 8B is involved in mitochondrial electron transport [34], and natriuretic peptide-53 is produced by endothelial cells. This peptide is thought to be very important in the regulation of local tissue and nerve conduction, and inhibition of cell proliferation [35]. Many factors can induce endothelial cells to secrete natriuretic peptide-53, including numerous growth factors and cytokines, such as tumor necrosis factor (TNF-α) and TGF-β [36]. In this study, the four significantly differentially expressed protein peaks in the treated cells all seem to be associated with regulating cell proliferation and cell signal transduction. Unfortunately, we did not detect changes in the levels of the proteins TGF-beta1, TIMP-1, TIMP-2, Col-1 and MMP2. The possible reason may be the inaccuracy of SELDI-TOF MS, where we did not obtain the mass of the protein fragments in mass spectrometric analysis. However, the data suggest that combined taurine, EGCG and genistein treatment may alter the expression of these proteins, thereby inhibiting HSC proliferation and contributing to the antifibrotic effects of the combined treatment.

## 4. Materials and Methods

### 4.1. Chemicals

Genistein (4′,5,7-trihydroxyisoflavone), taurine (2-amino ethane sulfonic acid), dithiothreitol (DTT), urea, glycine, 3-[(3-cholamidopropyl)dimethylammonio]propanesulfonate (CHAPS), sodium dodecylsulphate, thiourea, tributyl phosphine, ampholine pH 3.0–10.0, apotinin, acetonitrile (ACN), sodium acetate (NaAc), trifluoroacetic acid (TFA), hydroxyethyl piperazine ethanesulfonic acid, phosphate buffered saline pH 7.4 (PBS), high pressure liquid chromatography water, phosphoric acid (88%), and ethanol (95%) were purchased from Sigma Co. (Louis, MO, USA); Dulbecco’s modified eagle’s medium (DMEM) was obtained from Gibco Co. (Grand Island, NY, USA); fetal bovine serum was obtained from Hyclone Co. (Logan, UT, USA); TRIzol was purchased from Invitrogen Co. (Carlsbad, CA, USA); reverse transcription kits (Verso 1-Step RT-PCR Kit) was purchased from Fermentas Co. (Cambridge, Canada), and EGCG was obtained from Leshan Yujia Tea Science and Technology Development Co., Ltd. (Sichuan, China).

### 4.2. Cells and Treatments

HSC-T6 cells, which were purchased from the American Type Culture Collection (Manassas, VA, USA), were grown in DMEM supplemented with 10% heat inactivated FBS at 37 °C in a humidified 5% CO_2_ atmosphere. The cultures were passaged by trypsinization every 3 days.

### 4.3. 3-(4,5-Dimethylthiazol-2-yl)-2,5-Diphenyltetrazolium Bromide (MTT) Assay

HSC-T6 cells were seeded into 96-well plates (1 × 10^4^ cells/well) and split into one of four groups: (I) control cells; (II) 0.015 mg/mL taurine, 0.0175 mg/mL EGCG and 0.0035 mg/mL genistein; (III) 0.03 mg/mL taurine, 0.035 mg/mL EGCG and 0.007 mg/mL genistein; and (IV) 0.06 mg/mL taurine, 0.07 mg/mL EGCG and 0.014 mg/mL genistein. After 12 h, cells were incubated with the different drug combinations specified above. Twenty hours later, 20 μL of MTT was added to each well and the medium was removed after 4 h, then 150 μL of DMSO was added to each well to dissolve the dye for another 30 min. The absorbance of each well was then measured at 570 nm using a microplate reader (Thermo Co., Waltham, MA, USA). The experiments were performed in triplicate.

### 4.4. Reverse Transcription-Polymerase Chain Reaction (RT-PCR) Analysis of Fibrosis-Related Genes

HSC-T6 cells were seeded into 50-mL culture flask (5 × 10^4^ cells/flask). Approximately 24 h after culture, cells were treated with or without the different drug combinations specified above for 24 h. Total RNA was extracted by the Trizol-phenol-chloroform method. The first strand cDNA was synthesized by reverse transcription of total RNA using oligo(dT) primers and SuperScript II RNaseH-Reverse Transcriptase (Invitrogen, Carlsbad, CA, USA). For PCR analysis, 5 μg of total cellular RNAs was used. The PCR primers were designed according to the sequences in GenBank and were as follows: β-actin, sense: 5′-AAC CCT AAG GCC AAC CGT GAA AAG-3′, antisense: 5′-TCA TGA GGT AGT CTG TCA GGT-3′, 240 bp; *TGF-β1*, sense: 5′-ATG GTG GAC CGC AAC AAC-3′, antisense: 5′-TGA GCA CTG AAG CGA AAG C-3′, 329 bp; *Col-I*, sense: 5′-TGC CGT GAC CTC AAG ATG TG-3′, antisense: 5′-CAC AAG CGT GCT GTA GGT GA-3′, 462 bp; *TIMP-1*, sense: 5′-CAT GGT GAG CCT CTG TGG AT-3′, antisense: 5′-GTT CAG GCT TCA GCT TTT GC-3′, 393 bp; *MMP2*, sense: 5′-TGG AAG CAT CAA ATC GGA CTG-3′, antisense: 5′-GCA AAG GGC AAA CAA AGC A-3′, 527 bp; and *TIMP-2*, sense: 5′-CCA AAG CAG TGA GCG AGA A-3′, antisense: 5′-TCC CAG GGC ACA ATA AAG TC-3′, 262 bp. The PCR reactions were carried out in a 20 μL reaction volume with diluted cDNA samples. The final reaction concentrations were as follows: 10 pmol sense and antisense primers, 250 μM deoxyribonucleotide triphosphate (dNTP) mix; 10× PCR buffer; and 0.5 U/reaction Ex *Taq* DNA polymerase. PCR was carried out with an initial denaturation step at 94 °C for 5 min and a final extension step at 72 °C for 10 min in a cycler (Bio-Rad Co., Hercules, CA, USA). Amplification cycling conditions were as follows: for *Col-I* and *MMP-2*: 30 cycles at 94 °C for 30 s, 55 °C for 30 s, and 72 °C for 30 s; for β-actin, *TGF-β1*, *TIMP-1*, and *TIMP-2*: 30 cycles at 94 °C for 30 s, 52 °C for 30 s, and 72 °C for 30 s. After PCR amplification, 10 μL samples of each PCR product were separated on 2% agarose gel, stained with ethidium bromide, and visualized by UV illumination. Semi-quantitative analysis of the intensity of each PCR product was performed using SLB Mylmager (UVP Inc., Upland, CA, USA) and ImageQuant™ TL (Amersham Biosciences/GE Healthcare Co., Foster, CT, USA). The experiments were performed in triplicate.

### 4.5. Protein Extraction

HSC-T6 cells were seeded into 50-mL culture flask (5 × 10^4^ cells/flask). Approximately 24 h after culture, cells were treated with or without the different drug combinations specified above for 24 h. The cell pellets were collected, washed three times with ice-cold PBS, resuspended in 1 ml of lysis buffer (9 M urea, 4% CHAPS, 40 mmol/L Tris, 1% DTT, 0.8% ampholine pH 3~10, 0.002% apotinin), placed on ice and cells vortexed every five min for a total of 20 min. After centrifugation at 12,000 × *g* for 15 min, the supernatants of each sample were divided into several aliquots and stored at −80 °C until ProteinChip array profiling analysis was carried out.

### 4.6. ProteinChip Array Processing

The proteins of cell samples were analyzed on cation-exchange (CM10). CM10 was assembled into an 8-well bioprocessor (Bio-Rad, Hercules, CA, USA) and preactivated for 30 min with the appropriate buffers (100 mM Tris-HCl, pH 9.0, or 100 mM sodium acetate, pH 4.0). Next, 90 μL of the respective binding buffer (50 mM NaAC pH 4.0) was mixed with 30 μL of the protein extract, and incubated for 60 min with shaking at 200 rpm. After two washes with binding buffer and one quick rinse with HPLC grade water, the ProteinChip was removed from the bioprocessor and dried at room temperature. Then, the spots were loaded twice with 1 μL of a saturated solution of sinapinic acid (SPA) dissolved in 50% ACN/0.5% TFA (*v*/*v*). Air-dried for 5 min and then another 1 μL of SPA in solution was applied and allowed to air-dry. All steps were carried out at room temperature (18–20 °C).

### 4.7. SELDI-TOF MS Analysis

Mass/charge (*m*/*z*) spectra of proteins with affinity to the Weak Cation Exchanger surface were generated in a Ciphergen Protein Biology System (PBS-IIc, Fremont, CA, USA) plus TOF-MS Reader (Ciphergen Biosystems, Bio-Rad, Hercules, CA, USA). Data were collected by averaging the results of a total of 200 laser shots with an intensity of 180, a detector sensitivity of 8, a high mass to *m*/*z* 100 k and an optimization range of *m*/*z* 2–20 k. Mass accuracy was calibrated externally using the All-in-One peptide mass standard (Ciphergen Biosystems, Bio-Rad, Hercules, CA, USA) and SELDI-TOF-MS analysis was performed on the same day. The experiments were performed in triplicate.

### 4.8. Spectra Processing Using Ciphergen Protein Chip Software

Protein peaks of all sample spectra were clustered with the Ciphergen Express software, version 3.0 (Ciphergen Biosystems, Bio-Rad, Hercules, CA, USA), performing Expression Difference Mapping. All spectral data were normalized by total ion current after background subtraction. The range of peak masses was analyzed between *m*/*z* 2–20 k because the majority of resolved protein/peptides were found in this range. The molecular masses from *m*/*z* 0–2 k were excluded from analysis because they were mainly the signal noises of the energy absorbing molecule. The Biomarker Wizard (Ciphergen Biosystems, Bio-Rad, Hercules, CA, USA) was subsequently used to obtain peak detection and clustering across all spectra in the training set with the following settings: signal/noise (first pass): 5; minimum peak threshold: 20% of all; mass error: 0.3%; and signal/noise (second pass): 2 for the *m*/*z* 2–20 k mass range. Proteomic peak features that differed significantly between groups were identified using the Mann-Whitney nonparametric test.

### 4.9. Statistical Analysis

Statistical analysis was performed using SPSS 11.5 for Windows (SPSS, Chicago, IL, USA). One-way analysis of variance (ANOVA) was used to compare the means among different groups, and Tukey’s test was used for *post hoc* multiple comparisons. The data were presented as the mean ± standard error (S.E.). A *p*-value <0.05 was considered statistically significant.

## 5. Conclusions

Our study shows that combined drug treatment with taurine, epigallocatechin gallate and genistein can inhibit HSC proliferation, and impact fibrosis-related gene and protein expression. The antifibrotic effects of this drug combination may be due to its effects on the expression of fibrogenic genes.

## Figures and Tables

**Table 1:** Discriminatory proteomic features between control and treated cells.

Proteomic feature (mean *m*/*z* value)	AUROC	* Average intensity of combination drugs relative to control	*p value
2897.76	0.81	0.55	0.02
4656.91	0.83	1.51	0.02
5078.35	0.88	2.32	0.03
5361.62	0.83	0.27	0.02
5562.06	0.86	0.43	0.03
5645.97	0.81	0.47	0.03
5798.33	0.81	0.24	0.04
6141.22	0.80	0.35	0.02
6499.74	0.81	0.29	0.04
6623.38	0.80	0.33	0.04
6735.19	0.86	0.32	0.04
6896.73	0.86	0.23	0.04

AUROC, Area under the receiver operating characteristics curve;

* Mann-Whitney test.

**Table 2:** Differentially expressed proteins identified by SELDI-TOF-MS.

*m*/*z*	Accession No.	Protein name
6896.73	NP_899094.1	Whey acidic protein four disulfide core domain 15A
6623.38	NP_036899.1	Luteinizing hormone release inhibiting factor
5798.33	Q61839.1	Natriuretic peptide-53
4656.91	NP_031777.1	Cytochrome c oxidase subunit 8B
