# Correction: Yan, L., et al. Combined Taurine, Epigallocatechin Gallate and Genistein Therapy Reduces HSC-T6 Cell Proliferation and Modulates the Expression of Fibrogenic Factors. *Int. J. Mol. Sci.* 2013, *14*, 20543–20554

**DOI:** 10.3390/ijms22052343

**Published:** 2021-02-26

**Authors:** Yan Li, Ying Luo, Xuerong Zhang, Xing Lin, Min He, Ming Liao

**Affiliations:** 1 College of Animal Science and Technology, Guangxi University, Nanning 530004, China; liyan01091@163.com; 2 Medical Scientific Research Center, Guangxi Medical University, Nanning 530021, China; luoyingyjy@163.com (Y.L.); xuerongzhang86@126.com (X.Z.); linxingyk@163.com (X.L.); heminmin163@163.com (M.H.)

The authors regret that, during the preparation of our published manuscript “Combined taurine, epigallocatechin gallate and genistein therapy reduces HSC-T6 cell proliferation and modulates the expression of fibrogenic factors” [1], we submitted an incorrect version of Figure 2. Thus, Figure 2 should be replaced with the following figure (Figure 1).

The correction does not change the scientific conclusions of the article in any way. The authors apologize for any confusion this error may have caused to the readers.

## Figures and Tables

**Figure 1:**
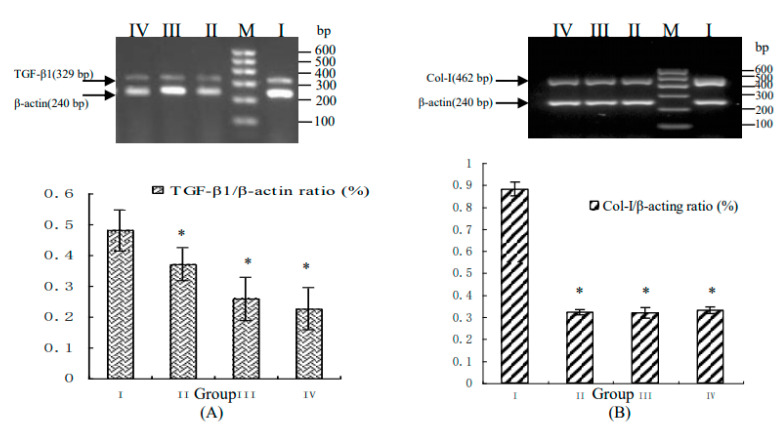

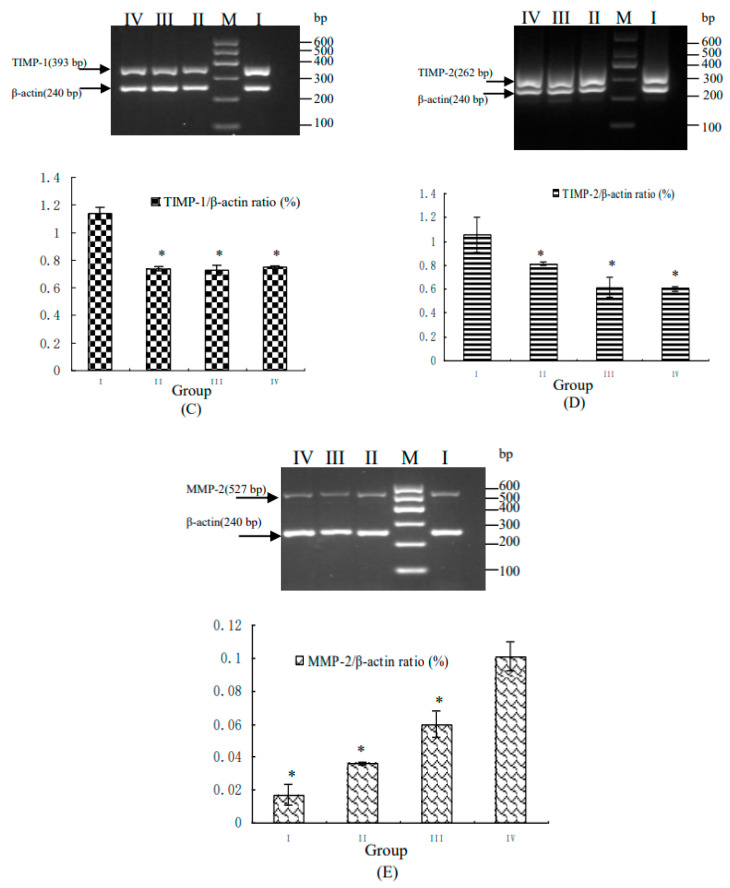
The mRNA expression levels of genes associated with hepatic fibrogenesis in HSC-T6 cells treated with a combination of taurine, EGCG and genistein. Semi-quantitative PCR data are shown for cells treated with a combination of taurine, EGCG and genistein in varying doses. The relative mRNA expression levels of *TGF-β1* (**A**), *Col-I* (**B**), *TIMP-1* (**C**), *TIMP-2* (**D**), and *MMP-2* (**E**) are presented; the upper panels show representative pictures of agarose gels and the lower panels show relative quantification of mRNA expression levels in (I) untreated control cells, or cells treated with (II) 0.015 mg/mL taurine, 0.0175 mg/mL EGCG and 0.0035 mg/mL genistein, (III) 0.03 mg/mL taurine, 0.035 mg/mL EGCG and 0.007 mg/mL genistein, or (IV) 0.06 mg/mL taurine, 0.07 mg/mL EGCG and 0.014 mg/mL genistein. (M) Marker. Data are presented as the mean ± S.E.; * *p* < 0.05 *vs.* group I (untreated control).
